# Puerperal Insanity

**Published:** 1865-08

**Authors:** 


### Puerperal Insanity.

The May number of the *Edinburgh Medical Journal* contains an interesting contribution to the statistics of Puerperal Insanity, as observed in the Royal Edinburgh Asylum, Morningside.

The writer, Dr. J. B. Tuke, has collected 155 cases of "so-called" puerperal mania, recorded during the last eighteen years in the case-books of the Asylum. He remarks that these cases comprise the more severe forms of the malady, inasmuch as those of a mild character are generally managed at home by the family medical attendant. The results of treatment of puerperal insanity, *as a whole*, therefore, are not attempted in this essay.

Dr. Tuke tabulates his cases under the following heads:—The Insanity of Pregnancy, Puerperal Insanity, and the Insanity of Lactation. Of the 155 cases, 28 belong to the first group, 73 to the second, and 54 to the third.

Dr. Tuke discusses at length the distinctive characteristics of these several groups, and the prognosis and treatment in each, and epitomizes the results in tabular form. The following are his conclusions:

1st—That an increase of liability to insanity exists between the ages of 30 and 40 in child-bearing women, and that first confinements occurring at that period are peculiarly frequently followed by true puerperal insanity.

2d—That Primiparæ are more commonly the subjects of the insanity of pregnancy and puerperal insanity than multiparæ.

3d—That the insanity of pregnancy in the majority of cases is developed during the third, fifth, or seventh months.

4th—That the insanity of pregnancy is generally evidenced by melancholia or moral perversion, and that it is very curable.

5th—That the hereditary tendency is peculiarly traceable in these three forms of insanity, and that in a large proportion of cases it exists on the female side of the family.

6th—That puerperal insanity leaves a tendency to other forms of insanity.

7th—That the puerperal insanity characterized by melancholia rarely commences until nearly a month after labor.

8th—That a tendency to suicide is a very frequent symptom.

9th—That complicated labors are more frequently followed by puerperal insanity than natural ones.

10th—That cases of puerperal insanity, in which acute mania is the leading symptom, are more amenable to treatment than those in which melancholia exists.

11th—That the insanity of lactation does not ensue on the first nursing so frequently as on subsequent ones, and the longer the child is kept to the breast the liability necessarily increases.

12th—That the insanity of lactation is more transient than either of the other forms, and that where evidenced by acute mania is less persistent than where melancholia exists.

13th—That delusions as to personal identity are very common symptoms in the three forms of insanity.

14th—That none of these forms of insanity are of themselves very fatal, except when complicated with other and especially inflammatory diseases. That they are all very amenable to treatment when such treatment is adopted early, and that the longer the patient is deprived of the benefits of an asylum the chances of recovery decrease.

15th—That this type of disease was anæmic in these three forms of insanity, which indicated the administration of a highly nourishing diet, but a very cautious use of stimulants.

16th—That the exhibition of narcotics is not beneficial where the leading symptom is acute mania.—*Am. Jour. Insanity.*

